# Removal of Copper (II) by Biochar Mediated by Dissolved Organic Matter

**DOI:** 10.1038/s41598-017-07507-y

**Published:** 2017-08-02

**Authors:** Pinjing He, Qinfang Yu, Hua Zhang, Liming Shao, Fan Lü

**Affiliations:** 1 State Key Laboratory of Pollution Control and Resource Reuse, Tongji University, Shanghai, 200092 China; 2 Institute of Waste Treatment and Reclamation, Tongji University, Shanghai, 200092 China; 3 Centre for the Technology Research and Training on Household Waste in Small Towns & Rural Area, Ministry of Housing Urban-Rural Development, Shanghai, 200092 China

## Abstract

The effects of humic acid (HA) and fulvic acid (FA) on Cu^2+^ adsorption on biochar were investigated, with mechanisms confirmed by excitation-emission matrix spectroscopy, Fourier transform infrared spectroscopy, and scanning electron microscopy. HA loading enhanced Cu^2+^ adsorption on biochar, with the maximum enhancement of 55.0% occurring at an HA loading of 100 mg-C/L. The adsorbed HA introduced many additional functional groups to biochar, thus enhancing Cu^2+^ adsorption, which decreased at HA concentrations >100 mg-C/L due to self-association of HA at high loading concentrations. In contrast, FA loading caused no enhancement on Cu^2+^ adsorption on biochar. FA was adsorbed through H-bonding with the functional groups of biochar, which set up a competition with Cu^2+^ for adsorption on biochar. The functional groups occupied by adsorbed FA were offset by the newly introduced functional groups of FA, thus there was no net increase in the amount of Cu^2+^ adsorption upon FA loading. These findings imply that, because of the enhanced adsorption of HA-loaded biochar, the amount of Cu^2+^ immobilized would increase by 28.2% for mature compost and 31.9% for fresh compost if there exist interaction between biochar and HA compared with the amounts immobilized by non-interactive HA and biochar.

## Introduction

Biochar is a carbon-rich product of the pyrolysis of biomass (i.e. plant materials including wood and crop residues or animal manure) under no or low oxygen conditions^1, 2^. Due to its low cost and potential ability to adsorb various contaminants^3, 4^, biochar has recently received much attention, especially for organic waste composting and remediation of soil or water contaminated by heavy metals^5^. Heavy metal contaminated matrices, such as organic waste, soil, sediment, and wastewater, are usually characterized by high contents of organic matter^6^. As a result, it would be useful to determine the selective preferences of biochar for adsorbing heavy metals versus organic matter. It has been reported that dissolved organic matter (DOM) is an important factor affecting the migration of heavy metals because it forms complexes with such metal ions^7, 8^, while at the same time biochar can interact with DOM upon contact^9, 10^. As a consequence, the presence of DOM might affect the removal efficiency of heavy metals by biochar. Unfortunately, to date, little information is available regarding interactions among biochar, DOM, and heavy metal ions.

There have been some studies about the effects of natural organic matter on the adsorption of heavy metal ions on other carbonaceous materials, such as active carbon^11, 12^, multiwalled carbon nanotubes and graphene oxide^13^. Compared with these materials, biochar generally exhibits less microporosity and a lower specific surface area^9, 14^ but contains more non-carbonized fractions^15^. Therefore, the adsorption mechanisms of biochar may be different from those of other carbonaceous materials.

In this study, we investigated the adsorption of Cu^2+^ on wood biochar in the absence and presence of humic acid (HA) or fulvic acid (FA). HA and FA are major components of naturally occurring DOM. Cu^2+^ was selected as a model heavy metal ion because it is commonly present in natural environments. Plant-derived biochar, produced at relative low temperatures, is rich in functional groups and has proved to be effective for heavy metal adsorption^16–18^. The objectives of the present study were to (1) investigate the effects of DOM on Cu^2+^ adsorption by biochar using two types of DOM (HA and FA) of different molecular sizes, aromaticity, and functional groups, which present different adsorption mechanisms on biochar and binding capacities for Cu^2+^, as well as (2) provide an insight into the mechanisms of such effects by observing changes in the functional groups and surface morphology of DOM-loaded biochar using Fourier transform infrared spectroscopy (FTIR) and scanning electron microscopy (SEM).

## Results

### Characterization of biochar, HA, and FA

SEM observations of the biochar, free-dried HA, and FA colloid are shown in Supplementary Fig. S1. The biochar had an open porous structure; the macropore size ranged from tens of nanometers up to tens of microns. HA displayed irregular morphology with different sizes within the range of tens of nanometers to several microns, indicating HA molecules easily formed agglomerates. FA colloid was relatively uniform with smaller sizes in the range of several to hundreds of nanometers.

Table 1 shows the characteristics of biochar and DOM. Biochar had a high C content of 69% on a dry weight basis, abundant O-containing functional groups of 1.20 mmol/g, and low specific surface area of 8.9 m^2^/g. Molar H/C and (O+N)/C ratios were used to indicate the aromaticity and polarity, respectively^19^. The H/C ratios of biochar, HA, and FA were 0.62, 1.19, and 1.84, respectively, indicating biochar had the highest aromaticity, and HA had higher aromaticity than FA, which was consistent with their specific ultraviolet absorbance at 254 nm (SUVA_254_) values (8.99 vs 2.29 L mg^−1 ^m^−1^). The higher (O+N)/C ratio of HA of 2.46 compared to that of FA of 1.25 implies HA contains more functional groups.

**Table 1:** Characteristics of biochar and DOM.

Sample	Elemental composition					Molar H/C	Molar (O+N)/C	Acid groups (mmol/g)	Specific surface area (m^2^/g)	SUVA_254_ (L mg^−1^ m^−1^)
	Ash	C	H	N	O					
	(%dw)									
Biochar	14.3	69.2	3.6	0.6	12.3	0.62 ± 0.01	0.14 ± 0.04	1.20	8.9	—
HA	14.4	19.6	2.0	0.1	64.0	1.19 ± 0.09	2.46 ± 0.11	—	—	8.99
FA	24.9	26.8	4.1	2.2	42.0	1.84 ± 0.07	1.25 ± 0.01	—	—	2.29

**Figure 1:**
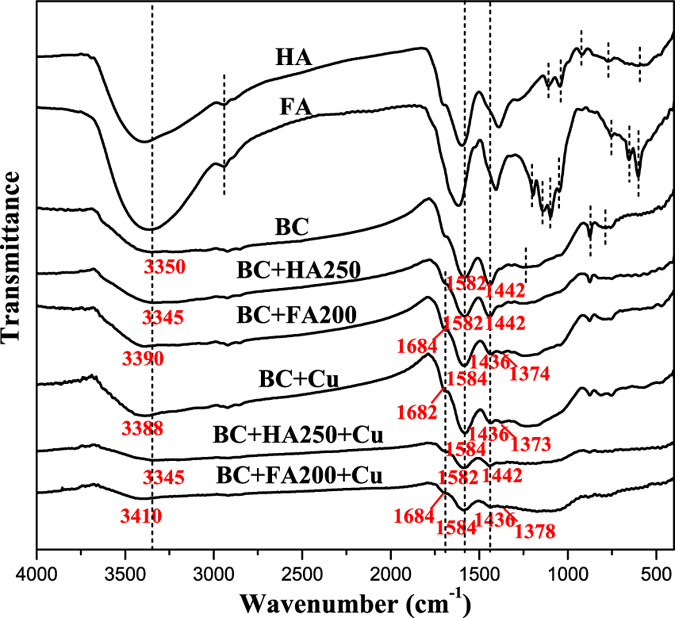
FTIR spectra of HA, FA, biochar, HA-loaded biochar, FA-loaded biochar, Cu-loaded biochar, Cu-HA-loaded biochar and Cu-FA-loaded biochar.

FTIR spectra (Fig. 1) show biochar has stretching vibrations of -OH and aliphatic -CH (represented by the peaks at 3350 and 2921 cm^−1^, respectively), a stretching vibration of aromatic C=O or C=C (1582 cm^−1^), -COO^−^ symmetric stretching (1442 cm^−1^), overlapping of aromatic CO- and phenolic -OH stretching of carboxylic acid (1250 cm^−1^), and aromatic -CH stretching (875 and 783 cm^−1^). HA has similar -OH, aliphatic -CH, aromatic C=O or C=C, and -COO^−^ stretching vibrations, (represented by the peaks at 3391, 2944, 1598, and 1389 cm^−1^, respectively), but additional aliphatic C-O-C vibrations (1110 and 1045 cm^−1^). FA has similar stretching vibrations to HA (peaks at 3364, 2942, 1616, and 1406 cm^−1^), but different C-O stretching vibrations of alcohol, ester, or polysaccharide groups (1196, 1140, 1097, and 1048 cm^−1^, respectively). FTIR spectra confirmed biochar has the highest aromaticity with fewer categories of functional groups, HA contains more aromatic rings while FA contains more aliphatic groups, characteristics that are consistent with their elemental compositions.

### Complexation between DOM and Cu^2+^

**Figure 2:**
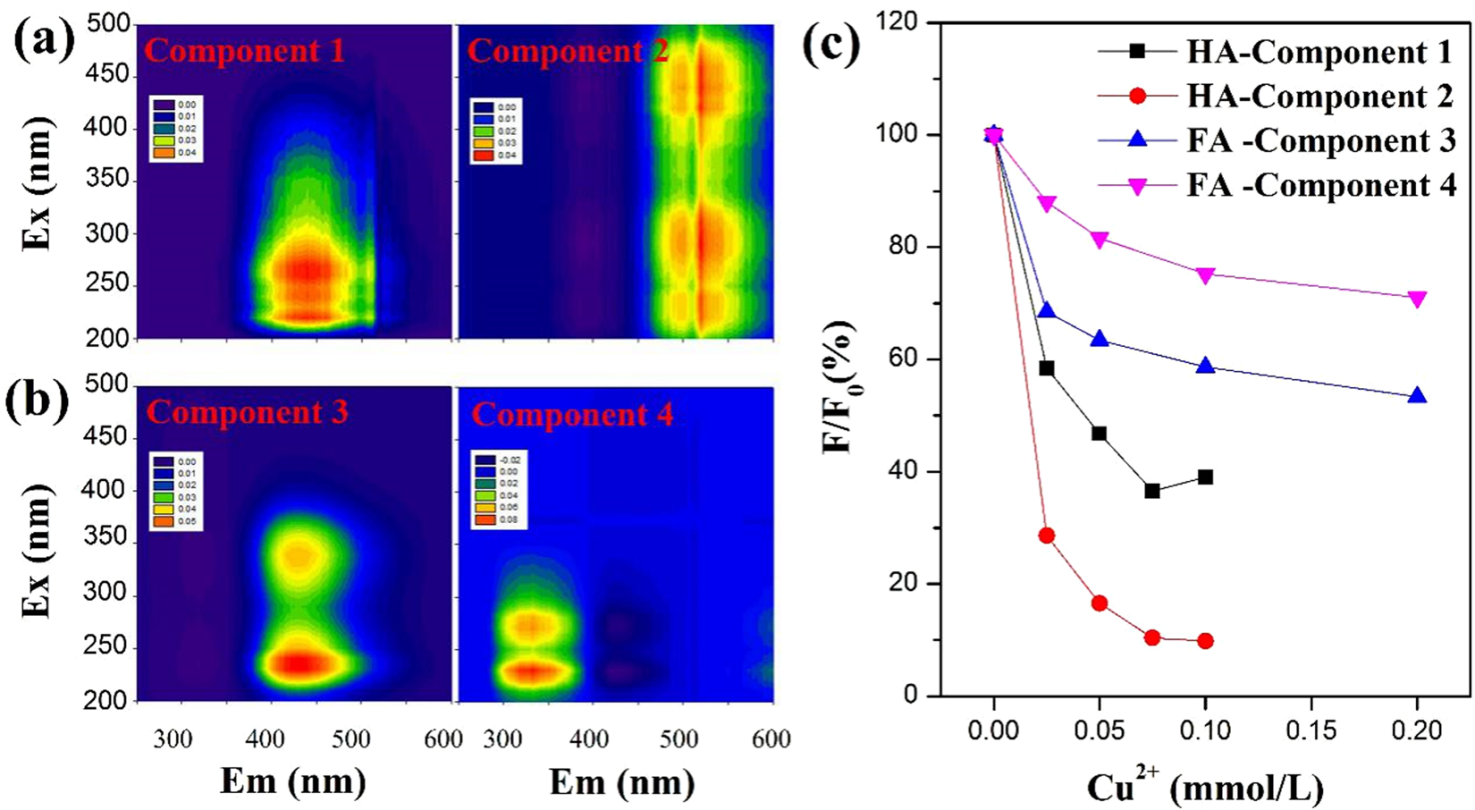
(**a**) PARAFAC-derived component of HA; (**b**) PARAFAC-derived component of FA; and (**c**) Fluorescence quenching of four PARAFAC-derived components with the addition of Cu^2+^. F/F0 means the ratio of fluorescence intensity with Cu^2+^ addition to the initial fluorescence intensity.

The fluorescence excitation-emission matrix (EEM) spectra (Supplementary Fig. S2) of the DOM solution with addition of Cu^2+^ indicate Cu^2+^ caused obvious fluorescence quenching of DOM. Using parallel factor (PARAFAC) analysis, EEMs related to HA can be decomposed into two humic-like components (component 1 at Excitation wavelength (Ex) 220, 260/Emission wavelength (Em) 440 nm and component 2 at Ex240, 290, 440/Em520 nm, Fig. 2a; the longer emission wavelength indicates component 2 might have a higher degree of humification than component 1^20^). EEMs related to FA can be decomposed into one humic-like component 3 at Ex230, 340/Em432 nm and one protein-like component 4 at Ex230, 270/Em332 nm (Fig. 2b). Figure 2c shows the quantitative analysis of fluorescence quenching for the four PARAFAC-derived components upon the addition of Cu^2+^. When the concentration of Cu^2+^ added reached 0.1 mmol/L, component 1 and component 2 of HA were quenched by 51.0% and 90.1%, respectively, and component 3 and component 4 of FA were quenched by 41.3% and 24.7%, respectively. The quenching of the four components displayed the following order: component 2> component 1> component 3> component 4, which is consistent with the order of their emission wavelengths, indicating components with a higher degree of humification were quenched more significantly^8, 21^. These results suggest that humic-like substances play an important role in the complexation between DOM and Cu^2+^; HA contains more aromatic humic-like components available for binding to Cu^2+^ than FA.

The binding of Cu^2+^ to HA and FA investigated by ion-selective electrodes is shown in Supplementary Fig. S3. The Langmuir equation was employed to fit the experimental data, and the binding constants obtained are given in Table 2. The binding capacity of HA for Cu^2+^ (222 mg-Cu/g-C-HA) was higher than that of FA (128 mg-Cu/g-C-FA), and FA had a higher affinity for Cu^2+^ (0.44 L/mg) than HA (0.33 L/mg). These results were consistent with the information about fluorescence quenching discussed above.

**Table 2:** Isotherm parameters for Cu^2+^ binding to DOM and adsorption on biochar.

Adsorbent	Langmuir			Freundlich		
	*q*_m_ (mg-Cu/g^[1]^)	*k*_L_ (L/mg-Cu)	R^2^	*k*_F_	n	R^2^
HA^[1]^	222 ± 13	0.33 ± 0.05	0.992	—	—	—
FA^[1]^	128 ± 11	0.44 ± 0.10	0.976	—	—	—
BC	18.8 ± 1.7	0.013 ± 0.005	0.880	2.3 ± 0.3	3.2 ± 0.3	0.980
BC-HA50	29.9 ± 1.9	0.009 ± 0.002	0.960	2.7 ± 0.5	2.8 ± 0.2	0.978
BC-HA250	19.5 ± 1.6	0.014 ± 0.004	0.893	2.5 ± 0.3	3.2 ± 0.2	0.985
BC-FA50	15.3 ± 1.1	0.016 ± 0.005	0.901	2.4 ± 0.3	3.6 ± 0.4	0.958
BC-FA200	16.9 ± 1.0	0.018 ± 0.005	0.921	3.0 ± 0.4	3.8 ± 0.3	0.978

[1]For HA and FA, g represents g-C.

The higher Cu^2+^ binding ability of HA can be attributed to its aromatic humic-like components, and in further detail ascribed to its greater quantity of functional groups and higher polarity^22, 23^, as indicated by the higher O content and (O+N)/C ratio of HA.

### Adsorption of DOM on biochar

**Figure 3:**
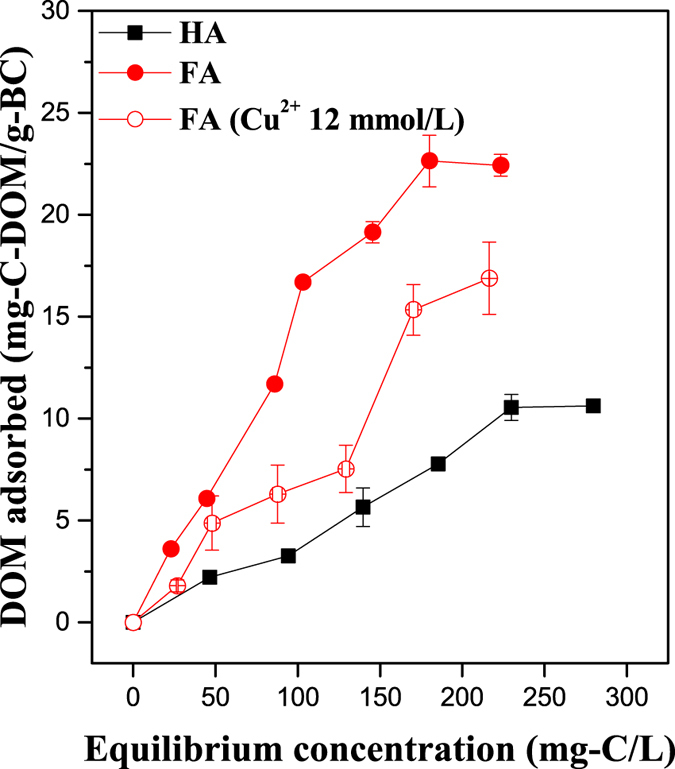
Adsorption isotherms of HA, FA, and FA in the presence of Cu^2+^ on biochar.

Adsorption isotherms of HA and FA on biochar are shown in Fig. 3. Conventional Freundlich and Langmuir models did not fit the isotherms well. Instead, the adsorption isotherms showed relatively high linearity (R^2^ = 0.94 for HA and 0.97 for FA), indicating partitioning of DOM contributed largely to adsorption amount, because the adsorption amount produced by the partition is proportional to the equilibrium concentration^24^. The adsorption capacities of biochar for HA and FA were about 10.6 mg-C-HA/g-BC and 22.5 mg-C-FA/g-BC, respectively.

The biochar used in the present study was not fully carbonized and thus contained non-carbonized and carbonized fractions, which could contribute to its adsorption behavior^24^. Partitioning on aliphatic C of the non-carbonized fraction may be one mechanism of DOM adsorption on biochar^24^. A pore-filling mechanism^25^ and hydrophobic interactions^26^ may also contribute to DOM adsorption. Biochar and DOM both have abundant π-electrons, so that π–π interactions may play an important role in DOM adsorption^25, 26^. H-bonding between the functional groups (-OH and -COOH) of biochar and DOM is another important mechanism^26^. Indeed, changes in the FTIR spectra of the biochars before and after DOM adsorption (Fig. 1) showed that the involvement of an H-bonding mechanism in FA adsorption was supported by shifts of the peaks relating to -OH (3350 cm^−1^ to 3390 cm^−1^) and -COOH (1582 cm^−1^ to 1584 cm^−1^, 1442 cm^−1^ to 1436 and 1374 cm^−1^, and the appearance of peak at 1682 cm^−1^), but such shifts were not observed for HA adsorption.

HA was adsorbed to a lesser extent on biochar than FA, mainly due to the larger size of HA as mentioned above and indicated in Supplementary Table S1. In addition, the macropore size of biochar ranged from tens of nanometers to tens of microns, so that size-exclusion behavior might cause some adsorbent sites on the biochar, such as micropores, mesopores, and functional groups, to be inaccessible to HA^27–29^.

Figure 3 also shows adsorption isotherms of FA in the presence of 12 mmol/L Cu^2+^. Adsorption isotherms of HA in the presence of Cu^2+^ were not obtained, for HA complexation with concentrations of Cu^2+^ higher than 0.1 mmol/L resulted in precipitation. The amount of adsorbed FA decreased by 20–50% in the presence of Cu^2+^, which suggests that the adsorptions of FA and Cu^2+^ on biochar are competitive. As discussed later, Cu^2+^ adsorption on biochar is mainly due to binding with the functional groups of biochar, so this competition confirmed again that FA adsorption on biochar occurs through H-bonding of the functional groups.

### Adsorption of Cu^2+^ on DOM-loaded biochar

Cu^2+^ adsorption isotherms on pristine biochar (BC), HA-loaded biochar (BC-HA50 and BC-HA250), and FA-loaded biochar (BC-FA50 and BC-FA200) are shown in Supplementary Fig. S4. Langmuir and Freundlich equations were employed to fit the adsorption isotherm data, and the adsorption constants and correlation coefficients are given in Table 2. The Freundlich equation (R^2^ = 0.96–0.99) fits the data slightly better than the Langmuir equation (R^2^ = 0.89–0.96). Biochar is heterogeneous both in its physical structure (e.g., porous structure) and chemical properties (e.g., carbon crystallinity). Therefore, it is reasonable that the Freundlich equation displays a better fitness for adsorption isotherm physical sorption^30^, since it assumes that reaction occurs at a heterogeneous surface, indicating Cu^2+^ adsorption on biochar is dominated by chemical sorption rather than physical sorption.

For Langmuir equation also fits the data well and provides the adsorption capacity, the following comparison will use the parameters fitted by the Langmuir equation to evaluate the adsorption capacity. The adsorption capacity of pristine biochar for Cu^2+^ was 18.8 mg-Cu/g-BC, within the reported range (6–90 mg-Cu/g-BC) of Cu^2+^ adsorption capacity for plant-based biochars^16, 17, 31, 32^. The adsorption capacity of the adsorbents for Cu^2+^ displays the following order: BC-HA50 > BC-HA250 ≈ BC > BC-FA200 > BC-FA50. Surface-loaded HA50 on biochar significantly enhanced the Cu^2+^ adsorption capacity to 29.9 mg-Cu/g-BC (paired t-test, p = 0.002), while the enhancement by loaded HA250 was very limited (19.5 mg-Cu/g-BC, paired t-test, p = 0.007).

**Figure 4:**
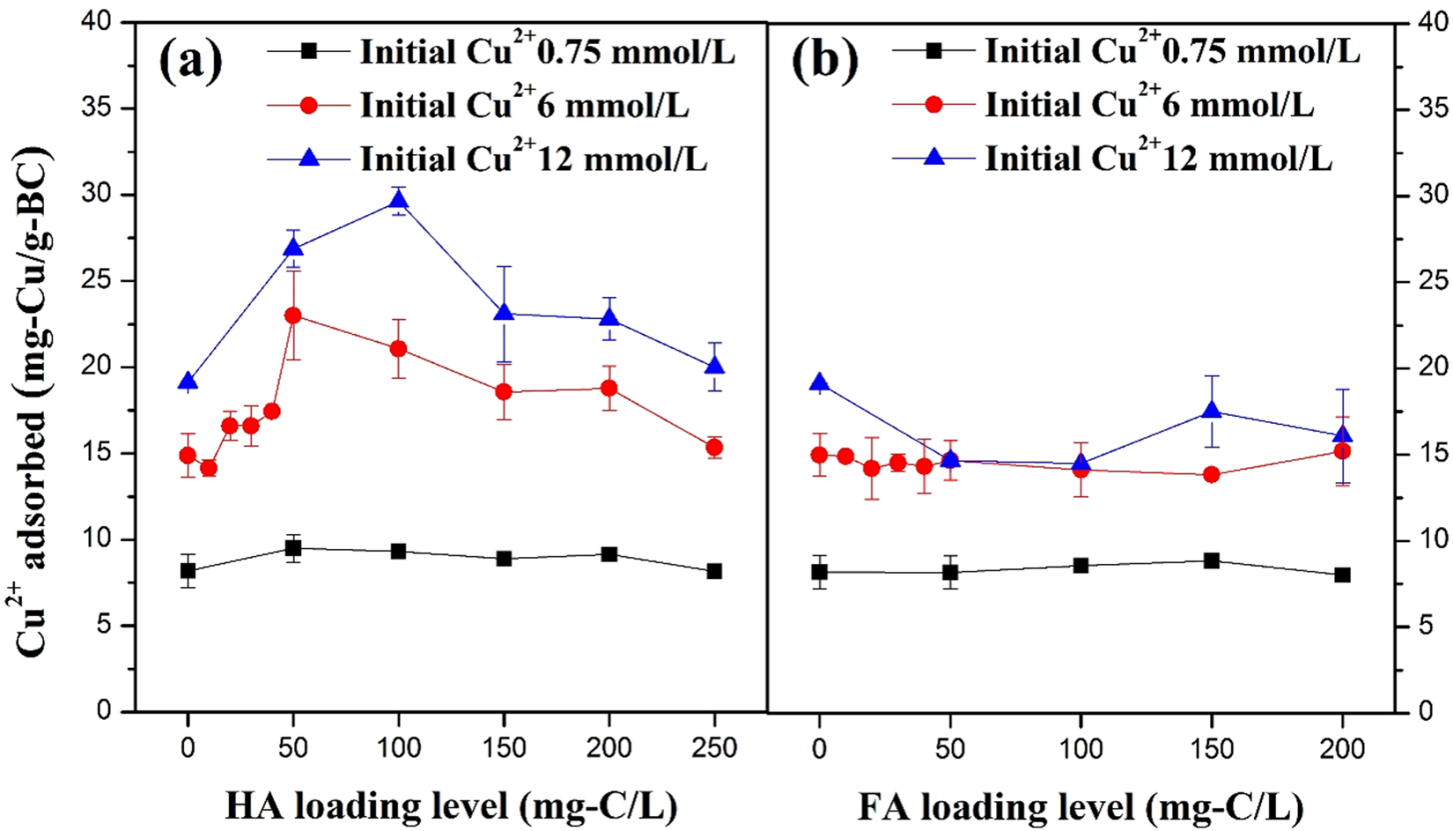
Effects of (**a**) HA loading level and (**b**) FA loading level on the amount of Cu^2+^ adsorption on biochar.

Figure 4a shows the effect of the HA loading level on the amount of Cu^2+^ adsorption at three initial Cu^2+^ concentrations. At the low initial Cu^2+^ concentration of 0.75 mmol/L, Cu^2+^ adsorption did not significantly increase with increasing HA loading levels (one-way ANOVA, p = 0.45). At higher Cu^2+^ concentrations of 6 mmol/L and 12 mmol/L, the amount of Cu^2+^ adsorption increased and then decreased as the HA loading level increased (one-way ANOVA, p = 0.001, 0.002, respectively), and the strongest adsorption occurred with a biochar loading of nearly 100 mg-C/L of HA.

Compared with HA, surface-loaded FA50 caused slight decreases in the Cu^2+^ adsorption capacity from 18.8 to 15.2 mg-Cu/g-BC (paired t-test, p = 0.005), surface-loaded FA200 had no significant effect on Cu^2+^ adsorption on biochar (paired t-test, p = 0.302), despite the larger amount of FA that was adsorbed on the biochar. Figure 4b shows that the amount of Cu^2+^ adsorption did not significantly increase at 0.75, 6 and 12 mmol/L Cu^2+^ with increasing FA loading level (one-way ANOVA, p = 0.69, 0.97 and 0.12, respectively).

### Stability of adsorbed Cu^2+^

Table 3 shows the amounts of Cu^2+^ adsorbed on BC, BC-HA50, BC-HA250, BC-FA50, and BC-FA200 at an initial Cu^2+^ concentration of 6 mmol/L, as well as the amounts extracted and extraction rates. All Cu^2+^ extraction rates were lower than 10%, which means Cu^2+^ adsorption on plant-derived biochar involved stable chemical binding. Moreover, Cu^2+^ adsorbed on BC-HA50 displayed the lowest extraction rate, suggesting strong binding of HA with Cu^2+^.

**Table 3:** Extractability of adsorbed Cu^2+^.

Sample	Cu^**2+**^ adsorption amount (mg-Cu/g-BC)	Cu^**2+**^ extraction amount (mg-Cu/g-BC)	Cu^**2+**^ extraction rate (%)
BC	14.9 ± 0.9	1.2 ± 0.1	8.2 ± 0.3
BC-HA50	23.0 ± 1.4	0.9 ± 0.1	4.0 ± 0.2
BC-HA250	17.3 ± 1.0	1.2 ± 0.1	6.8 ± 0.2
BC-FA50	14.6 ± 0.8	1.2 ± 0.0	8.3 ± 0.5
BC-FA250	15.3 ± 1.6	1.2 ± 0.1	8.1 ± 0.2

## Discussion

The ability of biochar to adsorb heavy metals is closely related to its characteristics, such as specific surface area, pore structure, and surface functional groups, which are determined by the raw materials and preparation conditions of biochar production^33, 34^. The biochar used in the present study is characterized by low specific surface area but an abundance of surface functional groups (e.g. -COOH and -OH). Therefore, Cu^2+^ adsorption on biochar could be attributed to the formation of surface complexes with functional groups^16, 31^, which can be demonstrated by changes in the FTIR spectra of biochars before and after Cu^2+^ adsorption (see Fig. 1). The complexation of functional groups with Cu^2+^ changes their chemical environment and thus leads to shifts of the peaks in the FTIR spectra^16, 35^. The -OH groups of biochar complexed with Cu^2+^, as evidenced by the shifts of the peaks at 3350 to 3388 cm^−1^. The -COOH groups of biochar also complexed with Cu^2+^, as evidenced by the changes in the peaks at 1682, 1582 and 1442 cm^−1^. These changes were similar to those observed for FA adsorption on biochar as mentioned above, indicating competitive adsorption between FA and Cu^2+^ on biochar.

Some studies have reported on the effects of organic matter on metal adsorption by highly carbonized materials, such as activated carbon and carbon nanotubes, which are usually characterized by high specific surface areas but low numbers of functional groups and are more likely to act by physical adsorption. Chen *et al*.^11^ and Terdkiatburana *et al*.^12^ found the presence of HA increased Cu^2+^ adsorption on active carbon, and increasing the HA concentration might increase or decrease or not affect the amount of Cu^2+^ adsorbed. Sheng *et al*.^22^ found HA enhanced Cu^2+^ adsorption on multiwalled carbon nanotubes at low pH values, and higher HA concentrations resulted in greater amounts of Cu^2+^ adsorption. Sun *et al*.^36^ found that different types of natural organic matter had different effects on Cu^2+^ adsorption by multiwalled carbon nanotubes, and the amount of Cu^2+^ adsorption increased with increasing natural organic matter concentrations. Yang *et al*.^37^ found that HA increased Cu^2+^ adsorption on few-layer reduced graphene oxide, but had little effect on Cu^2+^ adsorption on few-layer graphene oxide. These investigators showed that the effects of organic matter on metal adsorption by carbonaceous materials varied with the characteristics of the adsorbents and adsorbates, which determined the adsorption mechanisms.

**Figure 5:**
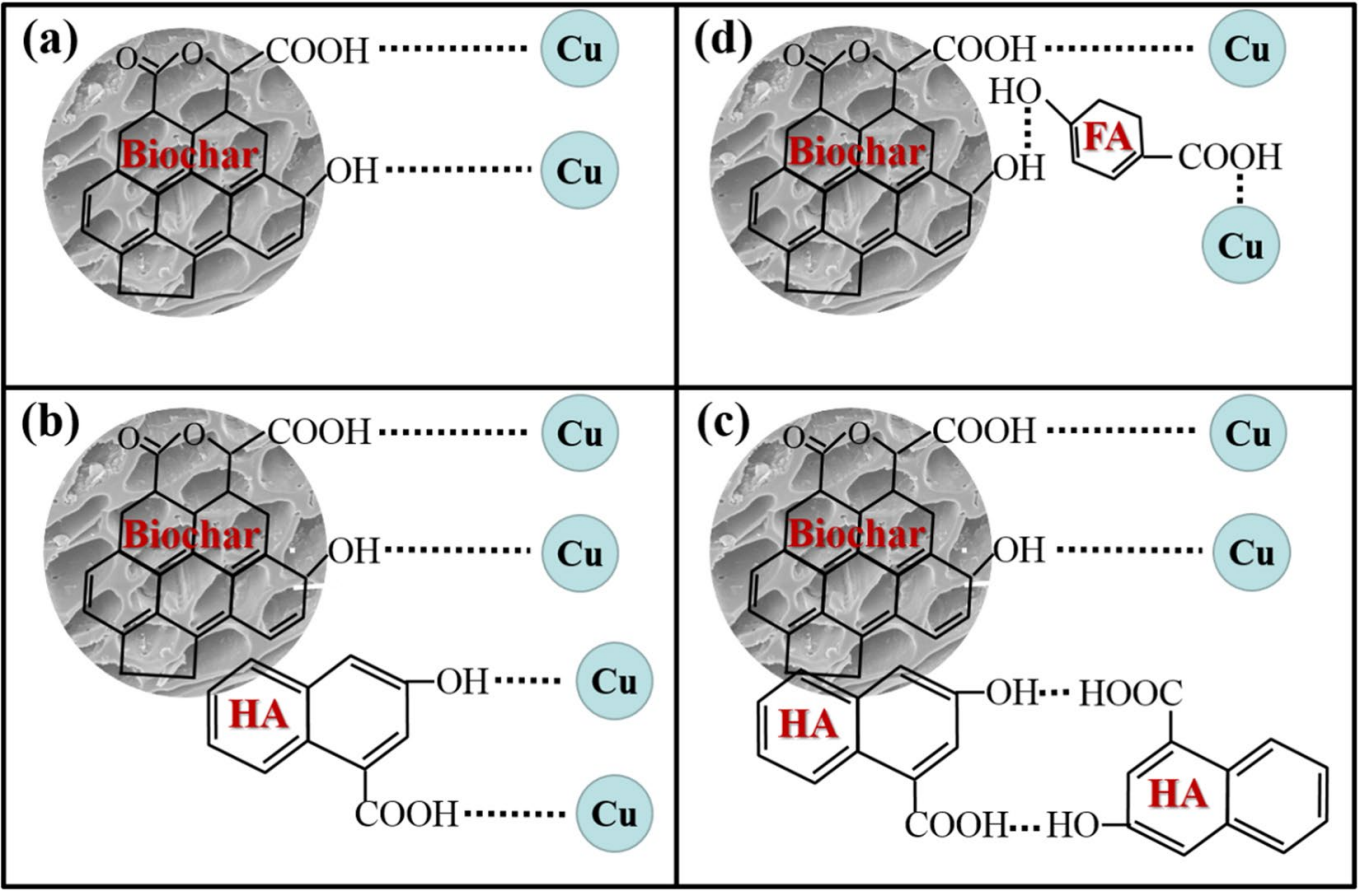
Schematic diagram of the interactions between Cu^2+^ and (**a**) biochar; (**b**) low levels of HA-loaded biochar; (**c**) high levels of HA-loaded biochar; (**d**) FA-loaded biochar.

In comparison, in this investigation, the potential interactions between Cu^2+^ and biochar loaded with DOM are shown in Fig. 5. As discussed previously, Cu^2+^ adsorption on biochar mainly occurred through binding with the functional groups (Fig. 5a), and HA was adsorbed on biochar mainly through partitioning and π–π interactions, not by complexing with the functional groups on biochar. Meanwhile, surface-loaded HA could introduce many additional functional groups through which biochar could bind to Cu^2+^, thus increasing the amount of Cu^2+^ adsorption. The amount of Cu^2+^ adsorption increased with increasing HA loading levels at HA concentrations <100 mg-C/L, because more functional groups were introduced (Fig. 5b). But a decrease was found with increasing HA loading levels at HA concentrations >100 mg-C/L, due to self-association of HA at high HA loading concentrations (Fig. 5c), which was supported by the SEM images of BC-HA50 and BC-HA250 (Supplementary Fig. S5). As shown in Supplementary Fig. S5, loaded HA50 was dispersed on the biochar surface, while loaded HA250 was agglomerated. Self-association occurs with high probability at high concentrations, especially for molecules such as HA of large molecular size^35^. HA self-associated via H-bonding between its functional groups^27^, thus making the functional groups unavailable for bonding to Cu^2+^. When the HA loading level reached 250 mg-C/L, nearly all functional groups of HA were self-bonding, which could explain the limited enhancement for loaded HA250. The above-mentioned studies have not noted this phenomenon for their carbonaceous materials^22, 23, 25–27^, perhaps due to lower organic matter concentrations of less than 100 mg-C/L and smaller molecular sizes than that in the present study.

As mentioned previously, FA was adsorbed on biochar mainly through H-bonding of functional groups. Obviously, the adsorbed FA molecules occupied functional group sites on the biochar and consequently decreased Cu^2+^ adsorption (Fig. 5d). Meanwhile, the absorbed FA also introduced functional groups for binding to Cu^2+^. These two effects occur simultaneously and caused a slight net decrease by surface-loaded FA50 (p = 0.005) or no significant effect by surface-loaded FA200 (p = 0.302) in the amount of Cu^2+^ adsorption.

The different effects of HA and FA could be attributed to their different characteristics. Compared with FA, HA has greater aromaticity, larger molecular size, and more functional groups, which resulted in a different adsorption mechanism on biochar and different binding capacity for Cu^2+^.

The present results indicate that the presence of DOM causes no large reduction in Cu^2+^ adsorption on biochar (only surface-loaded FA50 reduces Cu^2+^ adsorption by 19.1%). This implies that typical biochar, which has a low specific surface area and abundant surface functional groups, can serve as a remediation agent at DOM-rich heavy metal-contaminated sites.

Recently, biochar has been widely used as an amendment to the composting process of organic wastes such as sewage sludge and manure, which usually contain heavy metals that limit the application of compost to soil. Some researchers have found reduced mobility of heavy metals during composting, attributed to the production of HA that is available to form stable HA-metal complexes^38, 39^, and biochar addition has been found to further reduce the mobility of heavy metals in compost^40, 41^. According to the present results, calculations (described in detail in the Supporting Information) were performed to estimate the amounts of immobilized Cu^2+^ in fresh and mature compost (Table 4). Commonly, fresh sludge compost contains relatively higher amounts of FA and lower amounts of HA but, as composting proceeds, the FA content remains unchanged or slightly decreases while the HA content increases^38, 42, 43^. With the addition of 30% (dw/dw) biochar to the sludge compost, during the composting^34^, for per gram C of fresh and mature compost whose mass bases not account biochar, there exist 0.91 and 1.85 gram biochar. The present results show FA and HA can complex 128 mg-Cu/g-C-FA and 222 mg-Cu/g-C-HA, respectively. Because of the solubility of FA and insolubility of HA, the FA-Cu complex is generally regarded as being soluble, not immobilized^38^; hence fresh and mature compost can immobilize 6.7 and 22.2 mg-Cu/g-C-compost, mainly due to HA. Additional biochar alone can immobilize 18.8 mg-Cu/g-BC, namely 17.1 and 34.8 mg-Cu/g-C-compost in fresh and mature compost. (1) If biochar does not react with HA and FA, fresh and mature compost with biochar added can in total immobilize 23.8 and 57 mg-Cu/g-C-compost, respectively. (2) Assuming that biochar only adsorbs FA, FA-loaded biochar can immobilize 14.6 and 29.6 mg-Cu/g-C-compost in fresh and mature compost, and the sums are 21.3 and 51.8 mg-Cu/g-C-compost, respectively. (3) Assuming that biochar only adsorbs HA, in fresh compost, the remaining HA and HA-loaded biochar can respectively immobilize 6.1 and 25.3 mg-Cu/g-C-compost in fresh compost, in total 31.4 mg-Cu/g-C-compost. In mature compost, the remaining HA and HA-loaded biochar can respectively immobilize 20.7 and 52.4 mg-Cu/g-C-compost in fresh compost, in total 73.1 mg-Cu/g-C-compost. Therefore, owing to the enhanced adsorption of the HA-loaded biochar, the immobilized Cu^2+^ amount would increase by 28.2% for mature compost and 31.9% for fresh compost if interaction exist between biochar and HA when compared with non-interactive HA and biochar. Considering that biochar can further promote the humification of organic matter^34^, the metal-immobilization benefit from biochar addition during the biodegradation process may be more significant than expected.

**Table 4:** Potentially amounts of immobilized Cu^2+^ in fresh and mature compost.

Items (per g-C-compost)	Substance	Fresh compost	Mature compost
Content (g^[1]^)	FA^[1]^ in compost	0.08	0.08
	HA^[1]^ in compost	0.03	0.10
	Biochar	0.91	1.85
Alone immobilized Cu (mg-Cu): biochar doesn’t adsorb FA or HA	FA in compost	10.2 (soluble)	10.2 (soluble)
	HA in compost	6.7	22.2
	Biochar	17.1	34.8
	Sum1^[2]^	34.0	67.2
	Sum2^[3]^	23.8	57
Combined immobilized Cu (mg-Cu): biochar adsorbs FA only	FA in compost (adsorption deducted)	7.9 (soluble)	7.7 (soluble)
	HA in compost	6.7	22.2
	Biochar (loaded with FA)	14.6	29.6
	Sum1^[2]^	29.2	59.5
	Sum2^[3]^	21.3	51.8
Combined immobilized Cu (mg-Cu): biochar adsorbs HA only	FA in compost	10.2 (soluble)	10.2 (soluble)
	HA in compost (adsorption deducted)	6.1	20.7
	Biochar (loaded with HA)	25.3	52.4
	Sum1^[2]^	41.6	83.3
	Sum2^[3]^	31.4	73.1

^[1]^For HA and FA, g represents g-C.

^[2]^Sum1 means the sum of Cu^2+^ immobilized by FA, HA and biochar.

^[3]^Sum2 means the sum of Cu^2+^ immobilized by HA and biochar, where FA-Cu complex is regarded as not immobilized.

## Methods

### Preparation of biochar, HA, FA and DOM-loaded biochars

Commercial biochar (Qunfang Gardening, China) was obtained by pyrolysis of fruitwood at nearly 400 °C in kilns. It was crushed and sieved to 0.25–0.5 mm, washed with water and dried at 105 °C for 24 h prior to characterization and adsorption experiments. Commercial HA (Bomei Biotechnology, China) was dissolved in 0.1 mol/L NaOH and adjusted to pH 7.0 with HCl; commercial FA (Dibai Chemical Technology, China) was dissolved in water and adjusted to pH 5.0 with HCl. Both solutions were filtered with a 0.45-μm filter membrane, and the filtrates were retained as stock solutions and freeze-dried for subsequent analysis and experimentation.

DOM-loaded biochars were prepared by shaking biochar (2 g/L) in HA or FA solutions of various concentrations in a thermostatic shaker at 160 rpm and 25 °C for 8 d for HA loading and 5 d for FA loading. After loading with DOM, the biochar samples were collected on quantitative filter paper, and then washed via a continuous flow of water with a liquid-to-solid ratio of 200 mL/g and dried at 55 °C for 2 d. The obtained samples were named BC-HAn and BC-FAn, respectively. The number “n” represents the initial concentration of HA or FA in mg-C/L.

### Characterization of biochar, DOM, and Cu/DOM-loaded biochar

The point of zero charge (PZC) of biochar was determined by the pH drift method^44^, pH_PZC_ of biochar was 8.0 ± 0.1 (Supplementary Fig. S6), i.e., biochar had a net positive charge at the pH of 5.0 (<8.0) in the present Cu^2+^ adsorption experiments. The surface morphologies of HA, FA, biochar, and DOM-loaded biochar (BC-HA50, BC-HA250, BC-FA50, and BC-FA200) were characterized by SEM (S-4800, Hitachi, Japan), to observe the morphology changes of biochar after DOM adsorption. The specific surface area was characterized by N_2_ adsorption at 77 K using a specific surface area analyzer (ASAP 2020 M, Micromeritics Instrument, USA), and calculated by applying the Brunauer-Emmett-Teller equation. The elemental (C, H, N) compositions of biochar, HA, and FA were analyzed using an elemental analyzer (Vario EL III, Elementar, Germany). Ash content was measured by combusting the samples at 850 °C for 1 h, and the oxygen content was calculated by mass balance. The SUVA_254_ has been widely used to indicate the abundance of aromatic carbon in DOM^45^, measured using a UV spectrophotometer (UV-1800, Shimadzu, Japan) with a 1-cm quartz cell, and the SUVA_254_ values of HA and FA were calculated by dividing the absorbance at 254 nm by the carbon concentration. FTIR was used to analyze the chemical functional groups of biochar and DOM, as well as DOM/Cu-loaded biochar, to identify adsorption mechanisms; samples were mixed with KBr (sample: KBr = 3:100, W/W) and FTIR scans in the range 4000–400 cm^−1^ wavenumbers were accumulated (Nicolet 5700, USA). In addition to FTIR analysis, the O-containing acidic surface groups of biochar were quantitatively determined using the Boehm titration method^46^, Surface acidity was calculated under the assumption that 0.05 mol/L NaOH neutralizes acidic groups including carboxyl, lactonic, and phenolic groups.

In order to unravel the interactions among Cu^2+^, DOM, and biochar, the present study adopted five sets of experiments as shown in Supplementary Fig. S7, i.e. (1) complexation between DOM and Cu^2+^, (2) adsorption of DOM on biochar in the presence of Cu^2+^, (3) adsorption of DOM on biochar to obtain DOM-loaded biochars, (4) adsorption of Cu^2+^ on biochar and DOM-loaded biochars, and (5) desorption of Cu-loaded biochars to evaluate the stability of adsorbed Cu^2+^.

### Complexation between DOM and Cu^2+^

The complexation experiment was carried out by mixing HA or FA solution with Cu(NO_3_)_2_ solution to generate a series of samples, where the final DOM and Cu^2+^ concentrations were 10 mg-C/L and 0–0.2 mmol/L, respectively. The solutions were adjusted to pH 4.8–5.0 and shaken at 160 rpm for 24 h at 25 °C to ensure complexation equilibrium.

The EEM spectrum of an aliquot of the mixed solutions was analyzed to identify the structural changes of DOM after complexing with Cu^2+^, thus indicating the binding sites on DOM for Cu^2+^. The EEM spectra obtained were quantitatively analyzed by PARAFAC analysis using MATLAB 7.1 (Mathworks, USA) using the N-way toolbox version 3.10^47^. The EEM spectra of samples were obtained using a fluorescence spectrophotometer (Cary Eclipse, Varian, USA) in scan mode with an emission wavelength of 250 to 600 nm at 2-nm increments and an excitation wavelength of 200 to 500 nm at 10-nm increments.

An aliquot of mixed solutions was further analyzed to measure the free Cu^2+^ concentration to study the binding capacity of DOM for Cu^2+^. Free Cu^2+^ concentrations were determined by using a Cu^2+^ ion-selective electrode (PXSJ-216F, Shanghai Yidian, China) in 0.1 mol/L NaNO_3_. A standard curve relating electrode voltage to free Cu^2+^ concentration was obtained by determining the voltages of various solutions of Cu(NO_3_)_2_ of known concentrations.

### Adsorption experiments

The adsorption isotherms of DOM on biochar in the absence and presence of Cu^2+^ and the adsorption isotherms of Cu^2+^ on biochar and DOM-loaded biochar (BC, BC-HA50, BC-HA250, BC-FA50, BC-FA200) were determined. Note: adsorption isotherms of HA in the presence of Cu^2+^ were not obtained because HA complexation with Cu^2+^ caused precipitation.

The effects of DOM loading levels on the adsorption of Cu^2+^ by biochar were further detailed, where the initial Cu^2+^ concentrations were fixed at 0.75, 6, and 12 mmol/L and the concentrations of DOM loaded on biochar varied from 0 to 250 mg-C/L for HA and 0 to 200 mg-C/L for FA.

All of the aforementioned adsorption experiments were carried out in 150-mL conical flasks containing 0.1 g of biochar in 50 mL of solution, kept in a 160-rpm thermostatic shaker at 25 °C, the adsorption experimental conditions are presented in Supplementary Table S2. The pH was adjusted with 1 mol/L HCl or NaOH, aiming to avoid forming copper precipitates. Once adsorption equilibrium was attained, the solids and liquids were separated using quantitative filter paper, and the concentrations of DOM and Cu^2+^ in the filtrate were determined using a total organic carbon analyzer (TOC-V_CPH_, Shimadzu) and inductively coupled plasma optical emission spectroscopy (720ES, Agilent, USA), respectively. The amounts adsorbed were calculated by subtracting the equilibrium concentrations from the initial concentrations.

### Isotherm models

Langmuir (Eq. 1) and Freundlich (Eq. 2) equations were employed to evaluate and compare the adsorptions.

1 $${q}_{{\rm{e}}}=\frac{{q}_{m}{k}_{{\rm{e}}}{C}_{{\rm{e}}}\,}{1+{k}_{{\rm{L}}}{C}_{{\rm{e}}}}$$

where *q*_m_ represents the adsorption capacity (mg/g), *k*_L_ is the constant related to the adsorption affinity (L/mg).

2 $${q}_{{\rm{e}}}={k}_{{\rm{F}}}{{C}_{{\rm{e}}}}^{1/n}$$

where *k*_F_ is related to the adsorption capacity and adsorption intensity, n represents the adsorption intensity. *q*_e_ and *C*_e_ are the adsorption amount (mg/g) and equilibrium concentration (mg/L) of adsorbate, respectively.

### Evaluation of the stability of adsorbed Cu^2+^

To evaluate the stability of adsorbed Cu^2+^, Cu^2+^-loaded biochars (with or without DOM) were shaken with 0.01 mol/L CaCl_2_ (simulating soil background electrolyte) as a desorbing agent at 160 rpm and 25 °C for 3 h. The stability was expressed in extractability of adsorbed Cu^2+^.

All experiments were performed in duplicate and the average values are reported.

### Statistical analysis

Statistical analysis were performed using SPSS 22.0 software (IBM, USA). Paired t-tests were used to identify whether DOM loading had significant effect on Cu^2+^ adsorption on biochar, one-way ANOVA were used to identify whether DOM loading level had effect on the amount of Cu^2+^ adsorption, p ≤ 0.05 indicates that the data has a statistically significant difference.

## Electronic supplementary material

Supplementary Information
