# Supplementary material for: Removal of Copper (II) by Biochar Mediated by Dissolved Organic Matter
Source: Sci Rep. 2017 Aug 2;7:7091. doi: 10.1038/s41598-017-07507-y (PMC5541037; doi:10.1038/s41598-017-07507-y)
Supplement: Supplementary file 1 — Supplementary Information [file 41598_2017_7507_MOESM1_ESM.doc]

**Supplementary Information**

**Removal of Copper (II) by Biochar Mediated by Dissolved Organic Matter**

Pinjing He^1,2,3^, Qinfang Yu^1,2^, Hua Zhang^1,2^, Liming Shao^2,3^, Fan Lü^1,2,*^

**Supplementary Method**

**Determination of molecular size distribution of HA and FA.** Molecular size distribution of HA, FA were analyzed by the Ultrafiltration technique with membranes (Yadong Co., China) of different pore size cutoffs (10 kDa, 50 kDa, 100 kDa and 0.02 μm). 50 mg-C/L of HA or FA solution was put into a stirred 300 mL cell, permeated the membrane under pressure of 0.1 MPa provided by nitrogen gas, and the corresponding permeate and retentate were then collected to measure the concentration using a total organic carbon analyzer (TOC-V_CPH_, Shimadzu, Japan). The sample was separated into five fractions of different size, i.e., <10 kDa, 10–50 kDa, 50–100 kDa, 100 kDa–0.02 μm and 0.02–0.45 μm.

**Supplementary Calculation**

**Calculations for the amounts of immobilized Cu^2+^ in fresh and mature compost.** Commonly, in fresh sludge compost, FA content is 0.06-0.10 (assumed to be 0.08) mg-C/g-C-compost and HA content is 0.02-0.05 (assumed to be 0.03); while in mature sludge compost, FA content is 0.06-0.10 (assumed to be 0.08) mg-C/g-C-compost and HA content is 0.06-0.14 (assumed to be 0.10) g-C/g-C-compost. With the addition of 30% (dw/dw) biochar to the sludge compost, the dry weight loss is about 35% and the total C content of the sludge generally varies from an initial value of 33% to a final value of 25% (dw/dw) during the composting, so for per gram C of fresh and mature compost whose mass bases not account biochar, there exist 0.91 and 1.85 gram biochar. The present results show FA and HA can complex 128 mg-Cu/g-C-FA and 222 mg-Cu/g-C-HA, respectively. Because of the solubility of FA and insolubility of HA, the FA-Cu complex is generally regarded as being soluble, not immobilized; hence fresh and mature compost can immobilize 6.7 and 22.2 mg-Cu/g-C-compost, mainly due to HA. Biochar alone can immobilize 18.8 mg-Cu/g-BC, namely 17.1 and 34.8 mg-Cu/g-C-compost in fresh and mature compost. 1) If biochar does not react with HA and FA, fresh and mature compost with biochar added can in total immobilize 23.8 and 57 mg-Cu/g-C-compost, respectively. 2) Assuming that biochar only adsorbs FA, the present results show the Cu^2+^ adsorption capacities for different loading levels of FA-loaded biochar are nearly unchanged at 16 mg-Cu/g-BC. Therefore, FA-loaded biochar can immobilize 14.6 and 29.6 mg-Cu/g-C-compost in fresh and mature compost, and the sums are 21.3 and 51.8 mg-Cu/g-C-compost, respectively. 3) Assuming that biochar only adsorbs HA, in fresh compost, 30 mg-C-HA/0.91 g-BC corresponds to an HA loading concentration of 66 mg-C-HA/L in the HA adsorption on biochar of present study, and the HA adsorption amount is 2.5 mg-C-HA/g-BC according to the adsorption isotherms of HA on biochar and Cu^2+^ adsorption amount on HA-loaded biochar is 27.8 mg-Cu/g-BC. Hence, the remaining HA and HA-loaded biochar can respectively immobilize 6.1 and 25.3 mg-Cu/g-C-compost in fresh compost, in total 31.4 mg-Cu/g-C-compost. In mature compost, 100 mg-C-HA/1.85 g-BC corresponds to the an HA loading concentration of 110 mg-C-HA/L, and the HA adsorption amount is 3.7 mg-C-HA/g-BC according to the adsorption isotherms of HA on biochar and Cu^2+^ adsorption amount on HA-loaded biochar is 28.3 mg-Cu/g-BC. That is, the remaining HA and HA-loaded biochar can respectively immobilize 20.7 and 52.4 mg-Cu/g-C-compost in fresh compost, in total 73.1 mg-Cu/g-C-compost.

**Supplementary Table S1.** **Molecular size distribution of HA and FA.**

|  | <10 K | 10 -50 K | 50 -100 K | 100 K-0.02 μm | 0.02 μm - 0.45 μm |
| --- | --- | --- | --- | --- | --- |
| HA | 12.9% | 6.1% | 36.0% | 10.6% | 34.4% |
| FA | 86.9% | 1.6% | 0.4% | 11.1% | 0 |

**Supplementary Table S2. Adsorption experimental conditions.**

| Adsorbent | Adsorbate | Initial concentration | Equilibrium pH | Equilibrium time |
| --- | --- | --- | --- | --- |
| BC | HA | 0–300 mg-C/L | 7.8–8.0 | 8 d |
| BC | FA | 0–250 mg-C/L | 4.8–5.0 | 5 d |
| BC | FA+Cu**^2+^** | FA:0–250 mg-C/L  Cu**^2+^**:12 mmol/L |  |  |
| BC/  BC-HA50/  BC-HA250/  BC-FA50/  BC-FA200 | Cu**^2+^** | 0–12 mmol/L |  | 1 d |
| BC-  HA0-300 | Cu**^2+^** | 0.75, 6, 12 mmol/L |  |  |
| BC-  FA0-250 | Cu**^2+^** | 0.75, 6, 12 mmol/L |  |  |


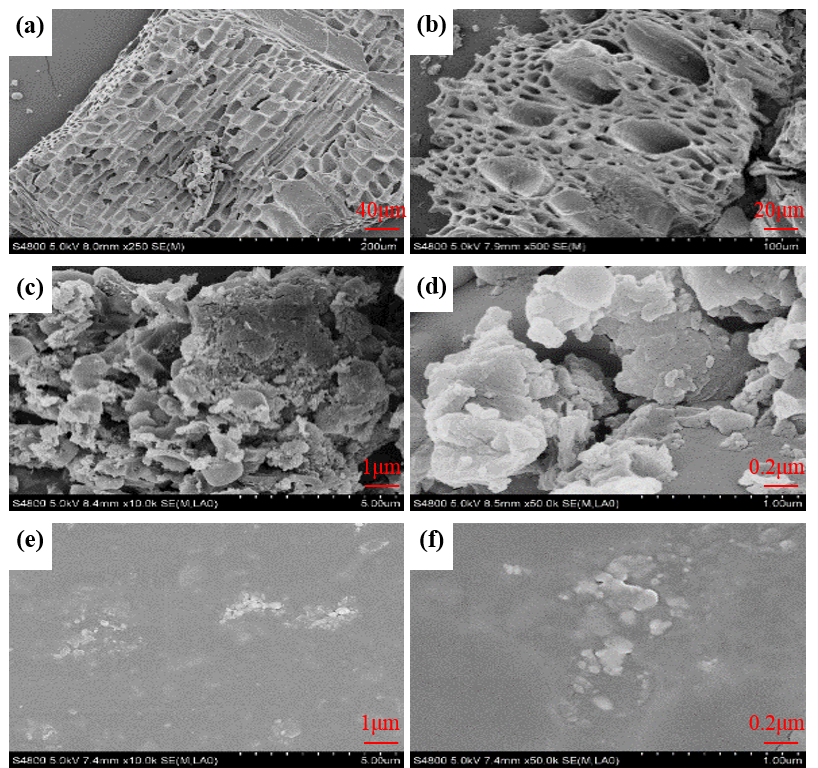
**Supplementary Fig. S1. SEM images of BC (a, b), HA (c, d), and FA (e, f).**

**Supplementary**
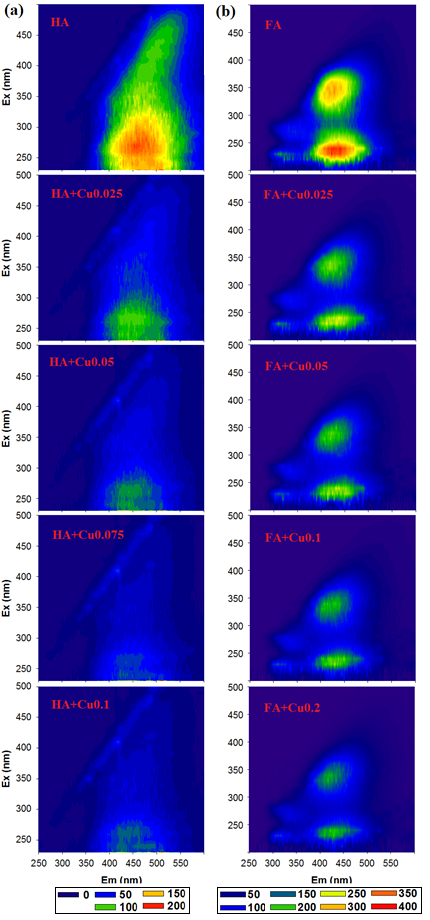
**Fig. S2. EEM spectra of (a) HA and (b) FA with addition of Cu^2+^** **of 0**–**0.2 mmol/L.**

**Supplementary Fig. S3. Binding of Cu^2+^ to HA and FA.**

**Supplementary Fig. S4. Cu^2+^ adsorption isotherms on biochar and DOM-loaded biochars.**


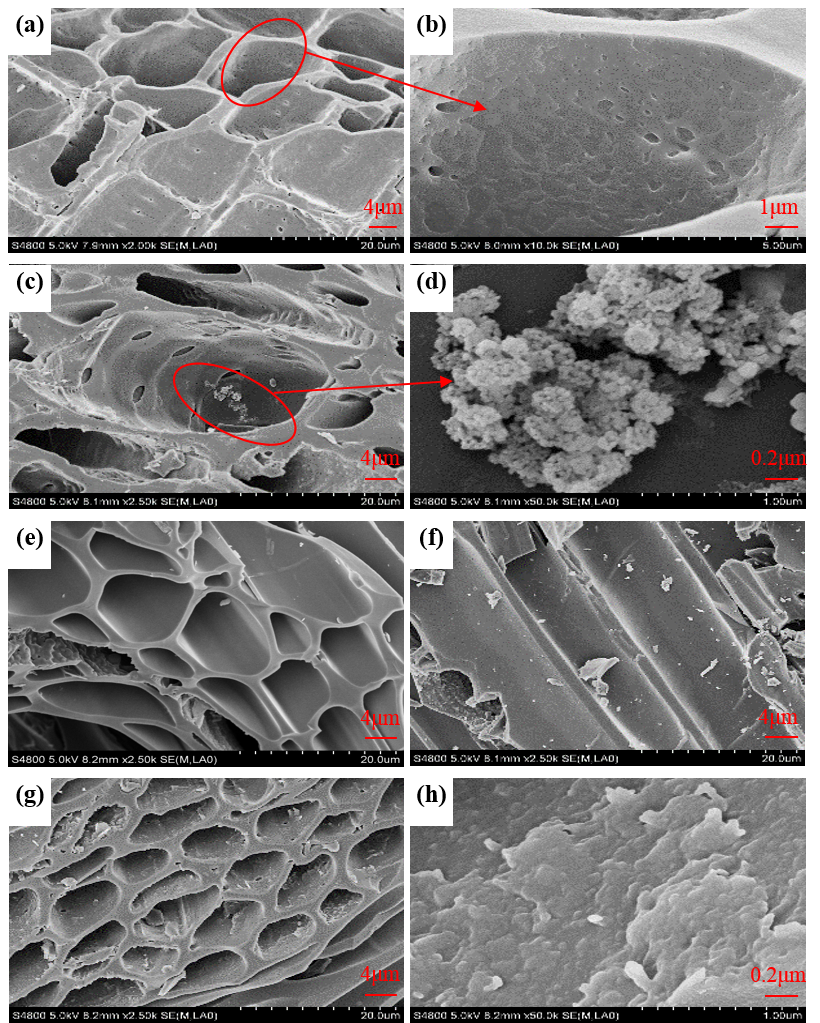
**Supplementary Fig. S5. SEM images of BC-HA50 (a, b), BC-HA250 (c, d), BC-FA50 (e, f), and BC-FA200 (g, h).**

**Supplementary Fig. S6. pH_PZC_ of biochar using the pH drift method. The pH at which pH_initial_ = pH_final_ was taken as** **pH_PZC_.**

**
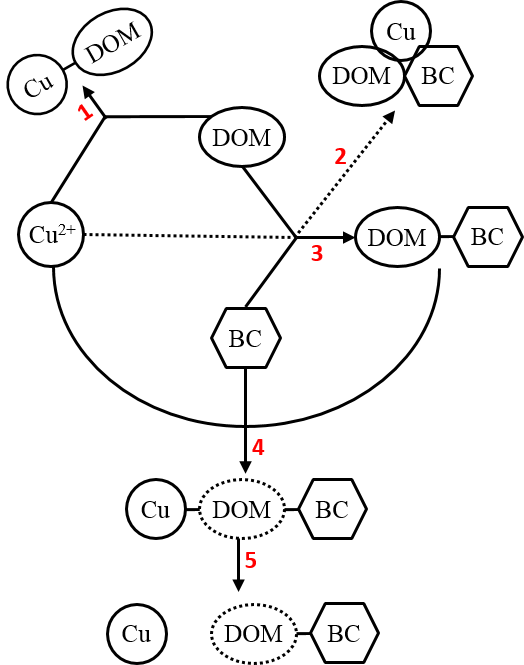
**

**Supplementary Fig. S7. Flow chart of experiments.** 1: Complexation between DOM and Cu^2+^; 2: Adsorption of DOM on biochar in the presence of Cu^2+^; 3: Adsorption of DOM on biochar and to obtain DOM-loaded biochars; 4: Adsorption of Cu^2+^ on biochar and DOM-loaded biochars; 5: Desorption of Cu-loaded biochars to evaluate stability of adsorbed Cu^2+^.
